# Deep-Sea Cold Seep Campylobacterota: Diversity, Growth, Metabolic Characteristics, and Nutrient Production

**DOI:** 10.3390/microorganisms13051028

**Published:** 2025-04-29

**Authors:** Xiaoman Yan, Qinglei Sun, Ke Xu, Jintao Zhuo, Yuanyuan Sun, Guowei Qian, Xin Zhang, Li Sun

**Affiliations:** 1 College of Life Science, Qingdao Agricultural University, Qingdao 266109, China; yanxm@qdio.ac.cn (X.Y.); xuke1552@163.com (K.X.); 2 CAS and Shandong Province Key Laboratory of Experimental Marine Biology, Center for Ocean Mega-Science, Institute of Oceanology, Chinese Academy of Sciences, 7 Nanhai Road, Qingdao 266071, China; sunyuanyuan@qdio.ac.cn (Y.S.); qianguowei@qdio.ac.cn (G.Q.); lsun@qdio.ac.cn (L.S.); 3 Laboratory for Marine Biology and Biotechnology, Laoshan Laboratory, Qingdao 266200, China; 4 Laoshan Laboratory, Qingdao 266237, China; zhuojintao@qdio.ac.cn (J.Z.); xzhang@qdio.ac.cn (X.Z.); 5 Key Laboratory of Ocean Observation and Forecasting, Key Laboratory of Marine Geology and Environment & Center of Deep Sea Research, Institute of Oceanology, Chinese Academy of Sciences, Qingdao 266071, China; 6 College of Marine Sciences, University of Chinese Academy of Sciences, Beijing 100049, China

**Keywords:** Campylobacterota, *Sulfurimonas*, *Sulfurovum*, cold seep, deep sea, vitamin

## Abstract

Deep-sea chemosynthetic ecosystems, including cold seeps and hydrothermal vents, are widely spread in global oceans. Campylobacterota are important primary producers in deep-sea hydrothermal vents and serve as a vital food source for local invertebrates. However, the nutrients that these bacteria can provide to their hosts are unclear. To date, research on Campylobacterota in cold seeps is very limited. Consequently, little is known about the biological features and ecological potential of Campylobacterota in cold seeps. In the present work, we examined the diversity, growth, metabolic characteristics, and nutrient production of Campylobacterota in a deep-sea cold seep. Over 1000 Campylobacterota ASVs, especially autotrophic *Sulfurovum* and *Sulfurimonas*, were identified. By optimizing the culture medium, 9 *Sulfurovum* and *Sulfurimonas* strains were isolated, including three potentially novel species. Two novel species were characterized and found to exhibit unique morphological features. These two novel strains possessed complete reverse tricarboxylic acid pathways. One novel strain, FCS5, was a psychrotolerant autotroph with denitrification and phosphorus-removing capacity. FCS5 could grow in the absence of vitamins. Consistently, metabolomics and transcriptome analyses indicated that FCS5 produced multiple vitamins, which regulated the expressions of a large number of genes associated with carbon fixation and multiple-nutrient synthesis. Besides vitamins, autotrophic Campylobacterota also produced abundant free amino acids, fatty acids (short-chain, medium, and long-chain), and proteins. This study indicates that the cold seep abounds with Campylobacterota, which are capable of providing various nutrients for the chemosynthetic ecosystem. In addition, these bacteria may have wide applications, such as in wastewater treatment and carbon emission reduction.

## 1. Introduction

According to the NCBI taxonomy, Campylobacterota (formerly known as Epsilonproteobacteria) presently encompasses Hippeaceae, Desulfurellaceae, Nautiliaceae, Nitratiruptoraceae, Hydrogenimonaceae, Sulfurovaceae, Arcobacteraceae, Sulfurimonadaceae, Helicobacteraceae, Sulfurospirillaceae, and Campylobacteraceae. Helicobacteraceae, Campylobacteraceae, Sulfurospirillaceae, and Arcobacteraceae are mainly heterotrophic, in particular *Helicobacter* and *Campylobacter*, which are common pathogens [1,2]. The other families, namely Hippeaceae, Desulfurellaceae, Nautiliaceae, Nitratiruptoraceae, Hydrogenimonaceae, Sulfurovaceae, and Sulfurimonadaceae, predominantly engage in a chemoautotrophic life relying on hydrogen gas or reduced sulfur, or follow a mixotrophic lifestyle [3]. Of the carbon-fixing families, members of Nautiliaceae, Nitratiruptoraceae, Hydrogenimonaceae, Sulfurovaceae, and Sulfurimonadaceae mainly utilize the rTCA (reverse tricarboxylic acid) pathway for carbon fixation [3], while Hippeaceae and Desulfurellaceae can employ the roTCA (reversed oxidative tricarboxylic acid) pathway [4]. Currently, the chemoautotrophic Campylobacterota that have been isolated are predominantly from the ocean, with hydrothermal vents—a typical chemosynthetic ecosystem—being a major source [5,6]. These isolated strains can be categorized into thermophilic and mesophilic types according to their growth temperatures, and a minor subset can even endure low temperatures [7,8,9]. These bacteria are extensively involved in the carbon, nitrogen, and sulfur cycles of hydrothermal vents [10,11]. In contrast, research on Campylobacterota in cold seeps, a different type of chemosynthetic ecosystem, is rather scant. Although a few reports indicate that autotrophic Campylobacterota is prevalent in cold seeps [12,13,14], only two strains of autotrophic Campylobacterota have been successfully isolated from cold seeps [15].

Autotrophic Campylobacterota have a crucial function as primary producers, and evidence indicates that these bacteria display extraordinary productivity in hydrothermal vents [16]. Autotrophic Campylobacterota frequently emerges as the dominant bacterial strains in hydrothermal vent plumes, sediment deposits, and microbial mats [17,18]. They are not only widely spread throughout the marine environment but also act as essential symbionts and have been found inside the cells (endosymbiosis) or on the surface (ectosymbiosis) of many invertebrates, such as snails (*Alviniconcha marisindica*), shrimp (*Alvinocaris longirostris*, *Rimicaris* spp.), and crab (*Shinkaia crosnieri*) [19,20,21,22,23]. In these holobionts, autotrophic Campylobacterota are known to be crucial nutrient providers. Previous research has indicated that *S. crosnieri* crab, which resides in hydrothermal vents, acquires nutrients by ingesting the autotrophic Campylobacterota attached to its setae [24]. A similar phenomenon was also reported in a cold seep, where lithodid crabs fed on the autotrophic Campylobacterota in microbial mats [13]. These observations suggest that autotrophic Campylobacterota may potentially serve as vital nutritional supplements for the host animals. This hypothesis is in line with the findings that autotrophic microbes in deep-sea chemosynthetic ecosystems are able to supply their host organisms with nutrients. For instance, hydrothermal vent-inhabiting tubeworms (vestimentiferans) undergo intestine atrophy during growth, which renders these worms wholly dependent on the symbiotic bacteria residing in their trophosome [25]. Once the trophosome bacteria disintegrate, copious amounts of nutrients, such as glycogen and vitamins, are released and taken up by the host, thus enabling the host’s rapid growth and expansion [26,27]. For autotrophic Campylobacterota, although known as symbiotic bacteria in chemosynthetic ecosystems, it is unclear what nutrients they can provide for their hosts.

The Formosa cold seep is situated on the continental slope of the South China Sea (SCS), with dimensions measuring approximately 100 m in both length and width. The seep’s central region features a hill-like elevation, serving as a habitat for a diverse assemblage of organisms, including *S. crosnieri* and *Alvinocaris* shrimp. Seawater within the animal community and the reduced sediments in this area exhibit elevated concentrations of methane (CH_4_) and hydrogen sulfide (H_2_S) [28]. In our prior research, a high abundance of Campylobacterota was identified [28]. Nevertheless, the overall diversity of Campylobacterota in cold seeps remains largely unexplored. In the present work, we investigated the diversity of Campylobacterota in the Formosa cold seep by employing both uncultured and cultured methodologies. Furthermore, since very few cold seep strains have been described hitherto, we isolated several novel strains and characterized their morphology, physiology, and metabolism. Finally, in order to explore the potential nutrients that might be provided by autotrophic Campylobacterota to the hosts, we analyzed the nutritional components of the isolated bacteria. The results of this study expand the knowledge of Campylobacterota in cold seeps and indicate that autotrophic Campylobacterota possesses the metabolic and physiological capacities to fulfill a vital ecological function in the food chain of the deep sea. This study also suggests an application potential of autotrophic Campylobacterota in nitrogen and phosphorus removal and carbon dioxide reduction.

## 2. Methods and Materials

### 2.1. Sampling, Medium Optimization, and Bacterial Enrichment and Isolation

Sediments were collected with a pushcore, and animal samples, including *Alvinocaris* shrimp and *S. crosnieri* crab, were collected with a television grab. Once aboard, the sediment was subsampled for every two centimeters and stored at −80 °C. The specimens were immediately washed thoroughly with sterile seawater, and the gills from *Alvinocaris* sp. and the setae from *S. crosnieri* were taken and stored at −80 °C. To optimize the medium for bacterial cultivation, the Sulfurimonas MJ medium (DSMZ 1011) was supplemented with various electron donors. The trace element solution and the vitamin solution were added according to DSM 140. The optimized medium was subsequently utilized to enrich autotrophic Campylobacterota from various samples. These enrichments were maintained at a temperature of 10 °C for 1 to 2 months. Bacterial isolation was accomplished through the method of serial dilution. The purity of the isolated strains was verified by means of 16S rRNA gene sequencing and heterotrophic culturing techniques.

### 2.2. Phenotypic, Phylogenetic, and Chemotaxonomic Analysis

For scanning electron microscope (SEM) analysis, the cells were first fixed with 2.5% glutaraldehyde in PBS (pH 7.4) for 2 h, followed by dehydration in a series of ethanol solutions at 4 °C. The dried cells, obtained using a critical point dryer (Hitachi-HCP, Hitachi, Tokyo, Japan), were then sputter-coated with platinum (MC1000, Hitachi, Tokyo, Japan) and examined with SEM (ZEISS Gemini-500, ZEISS, Oberkochen, Germany). For transmission electron microscope (TEM) analysis, the cells were initially fixed with 2.5% glutaraldehyde in PBS for 2 h and then washed three times (30 min/time) in 0.1 M PBS at 4 °C. Next, the cells were post-fixed with 1% osmium tetroxide in PBS for 1.5 h and washed as above. Subsequently, the samples were dehydrated in alcohol, infiltrated with a mixture of acetone and epoxy resin, and embedded and polymerized within epoxy resin. Ultrathin sections were prepared using a Leica EM UC7 ultramicrotome (Leica Microsystems, Nussloch, Germany) and transferred onto copper grids coated with the Formvar membrane. Contrast staining was performed using 2% uranyl acetate and lead citrate (Ted Pella INC, Redding, CA, USA). The sections were photographed with a TEM (HT7700, Hitachi, Tokyo, Japan).

Gram staining was carried out using a Gram staining kit (from Haibo, Qingdao, China). To investigate the temperature range of growth, the bacteria were cultivated at temperatures of 0, 5, 10, 15, 20, 25, 30, and 35 °C for three weeks, during which the OD_600_ value of the bacterial solution was measured. To examine the NaCl concentrations suitable for growth, the bacteria were grown in a medium containing different concentrations of NaCl (ranging from 0 to 10% *w*/*v*, with an increment of 1%) at the optimal temperature. To determine the pH range for growth, the bacteria were cultured at the optimal temperature in the presence of different pH buffers [29]. The doubling time of the bacteria was evaluated, as reported previously [30].

Bacterial utilization of electron donors was examined in the MJ medium with an N_2_/CO_2_ atmosphere. The following potential electron donors were tested: Na_2_S at the concentrations of 10, 20, 40, and 100 μM; Na_2_S_2_O_3_ at the concentrations of 5, 10, 20, 40, and 80 mM; Na_2_SO_3_ at the concentration of 5 mM (stocked in 2 mM Na_2_EDTA); Na_2_S_4_O_6_ at the concentration of 5 mM; and elemental S (S^0^) at the concentration of 5 g/L. For the test involving hydrogen as the electron donor, a gas mixture of 80% H_2_/20% CO_2_ was employed. The utilization of electron acceptors was investigated in the MJ medium with an 80% H_2_/20% CO_2_ atmosphere. The following potential electron acceptors were tested: NaNO_3_ at the concentration of 2 g/L; S^0^ at the concentration of 5 g/L; Na_2_S_2_O_3_ at the concentration of 5 mM; and O_2_ at the concentrations of 1%, 3%, 5%, and 7% (*v*/*v*). Heterotrophic growth was evaluated in the MJ medium without NaHCO_3_ under 100% N_2_, with the following organic substances being utilized: (a) glucose at the concentration of 0.1 mM; (b) a mixture of lactate, malate, fumarate, succinate, glycerine, and glucose at the concentration of 100 μM each; (c) yeast extract at the concentration of 0.01 g/L; (d) pyruvate at the concentration of 100 μM; (e) acetate at the concentration of 100 μM; (f) fumarate at the concentration of 100 μM; (g) an alcohol mixture (comprising butanol, ethanol, methanol, and propanol) at a concentration of 100 μM each component; and (h) peptone at the concentration of 0.01 g/L.

Phylogenetic analysis was carried out as reported previously [31]. For the analysis of fatty acid methyl esters (FAME), the bacteria were cultured at the optimum temperature and harvested at the late logarithmic stage. The data for FAME were analyzed as previously described [32].

### 2.3. Amplicon Analysis, Genomics, and Transcriptomics

Amplicon sequencing and analysis were carried out following the procedures of a previous report [28]. The universal primer set 341F (5′-CCTAYGGGRBGCASCAG-3′) and 806R (5′-GGACTACNNGGGTATCTAAT-3′) was used for amplification of the V3–V4 regions of 16S rRNA gene. Bacterial genome sequencing and analysis were conducted, as reported previously [31]. The functions of the genes were annotated by referring to the NCBI-NR and KEGG databases. Transcriptomic analysis was carried out at Novogene Bioinformatics Institute (Beijing, China). The details of the analysis are provided in the Supplemental data.

### 2.4. Chemical Composition Analysis

Nitrate (NO_3_–N), nitrite (NO_2_–N), ammonium (NH_4_–N), and phosphate (PO_4_^3−^) were determined with a Seal QuAAtro 39 autoanalyzer (Seal Analytical GmbH, Norderstedt, Germany). The Raman insertion probe (RiP) system was used to collect the Raman spectra of the gas (H_2_, N_2_, and CO_2_) above the liquid medium in the Hungate tubes [33].

### 2.5. Protein Content and Metabolomics Analysis

The protein content of microbial cells was determined using the Kjeldahl method. The sample was transferred into a digestion tube. Subsequently, 0.04 g of copper sulfate, 0.6 g of potassium sulfate, and 2 mL of sulfuric acid were added into the tube, followed by digestion for 1 h at 420 °C in a digestion furnace. The sample was then cooled, and 5 mL of water was added. The total nitrogen was measured with a fully automatic Kjeldahl nitrogen analyzer (Foss, Hilleroed, Denmark). The metabolomic analysis was carried out by Shanghai Applied Protein Technology Co., Ltd. (Shanghai, China), and the detailed information was described in the Supplemental data.

## 3. Results

### 3.1. Diversity of Campylobacterota in the Formosa Cold Seep of the South China Sea (SCS)

**Figure 1:**
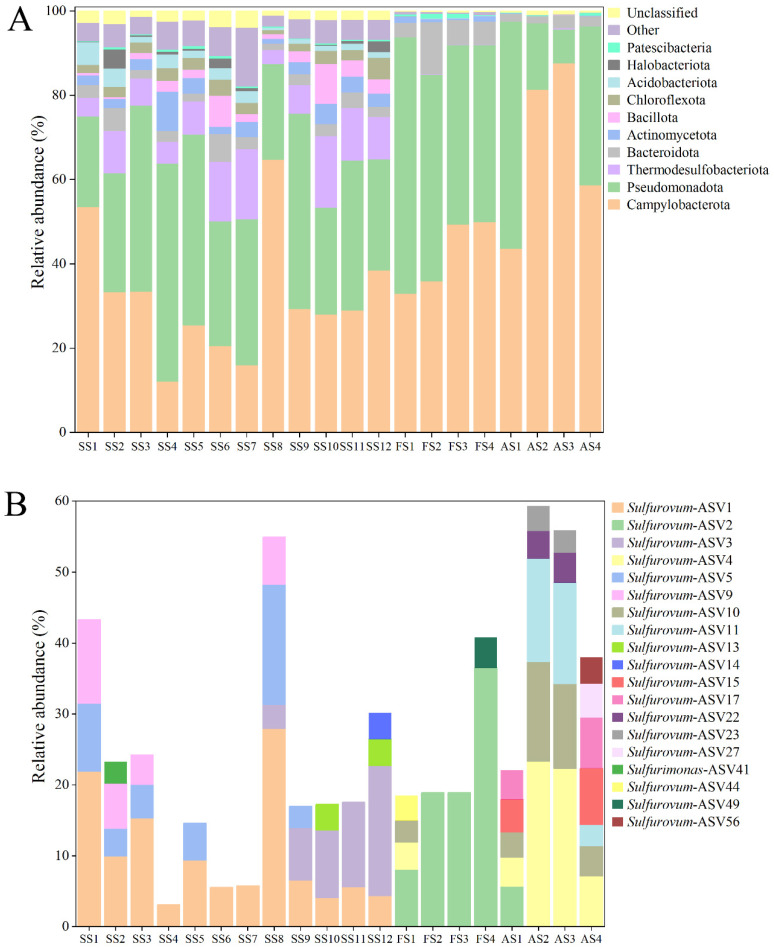
Diversity and distribution of microbial sequence tags in the sediment samples and animal samples. (**A**) The sequence tags were classified at the phylum level. Each color represents the percentage of the taxon in the total assemblage. The top 10 phyla are shown. (**B**) The Campylobacterotal ASVs had >3% abundance in at least one sample.

Samples were obtained from the sediment and animals (*Shinkaia crosnieri*) in the Formosa cold seep of SCS. The sediment sample (SS) was 24 cm in length and equally partitioned into 12 layers, which were designated SS1 (0–2 cm), SS2 (2–4 cm), SS3 (4–6 cm), SS4 (6–8 cm), SS5 (8–10 cm), SS6 (10–12 cm), SS7 (12–14 cm), SS8 (14–16 cm), SS9 (16–18 cm), SS10 (18–20 cm), SS11 (20–22 cm), and SS12 (22–24 cm), respectively. The animal-associated samples were obtained from *S. crosnieri* foot setae (named FS1 to FS4) and abdominal setae (named AS1 to AS4). All samples were subjected to microbial community analysis. A total of 1,239,094 tags were acquired (Table S1). For each sample, the number of tags ranged from 47,370 to 78,714, with a 100% coverage rate (Table S1). These results indicated that the sequencing depth was sufficient. The ASV ranges of the sediment and animal samples were 1166–2587 and 131–361, respectively (Table S1). Campylobacterota was abundant, accounting for 12.2–53.6% and 33.0–87.7% in the sediment and animal samples, respectively (Figure 1A). Campylobacterota comprised 1091 ASVs or 225 OTUs (97%) and belonged to six genera, namely *Sulfurovum*, *Sulfurimonas*, *Sulfurospirillum*, *Helicobacter*, *Campylobacter*, and *Arcobacter* (Table S2). A total of 98 ASVs and 37 OTUs could not be classified at the genus or family level (Table S2). *Sulfurovum* and *Sulfurimonas* exhibited the highest diversities, with 793 ASVs/140 OTUs and 154 ASVs/35 OTUs, respectively. At the ASV level, 18 *Sulfurovum*-ASVs were abundant (exceeding 3% in at least one sample), and one *Sulfurimonas*-ASV was abundant (Figure 1B). Specifically, *Sulfurovum*-ASV1 was abundant in all layers of the sediment, and *Sulfurovum*-ASV3 was abundant in the lower layers of the sediment; *Sulfurovum*-ASV2 had a high abundance in *S. crosnieri* foot setae, and *Sulfurovum*-ASV4/10/11 were plentiful in *S. crosnieri* abdominal setae (Figure 1B).

### 3.2. Enrichment and Isolation of Sulfurovum and Sulfurimonas

**Figure 2:**
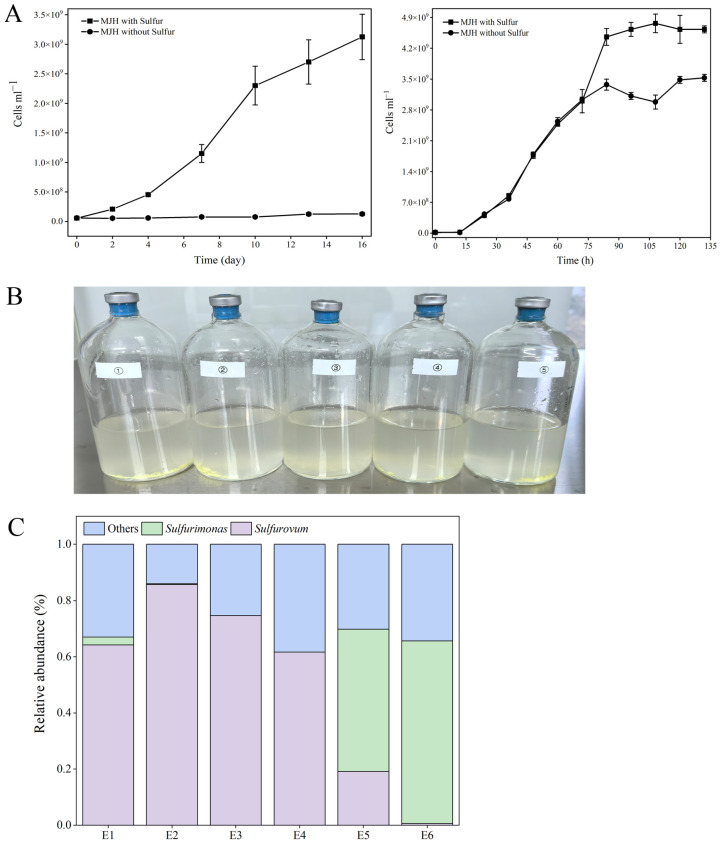
Medium optimization and enrichment of autotrophic Campylobacterota. (**A**) Cell counts of *Sulfurovum fonticola* CS14 (**left**) and *Sulfurimonas fonticola* CS47 (**right**) cultured in MJH medium with or without sulfur. Values are the means of triplicate experiments and shown as means ± SD. (**B**) Representatives of the enrichments. (**C**) Distribution of *Sulfurovum* and *Sulfurimonas* in different enrichments (E1 to E6).

**Figure 3:**
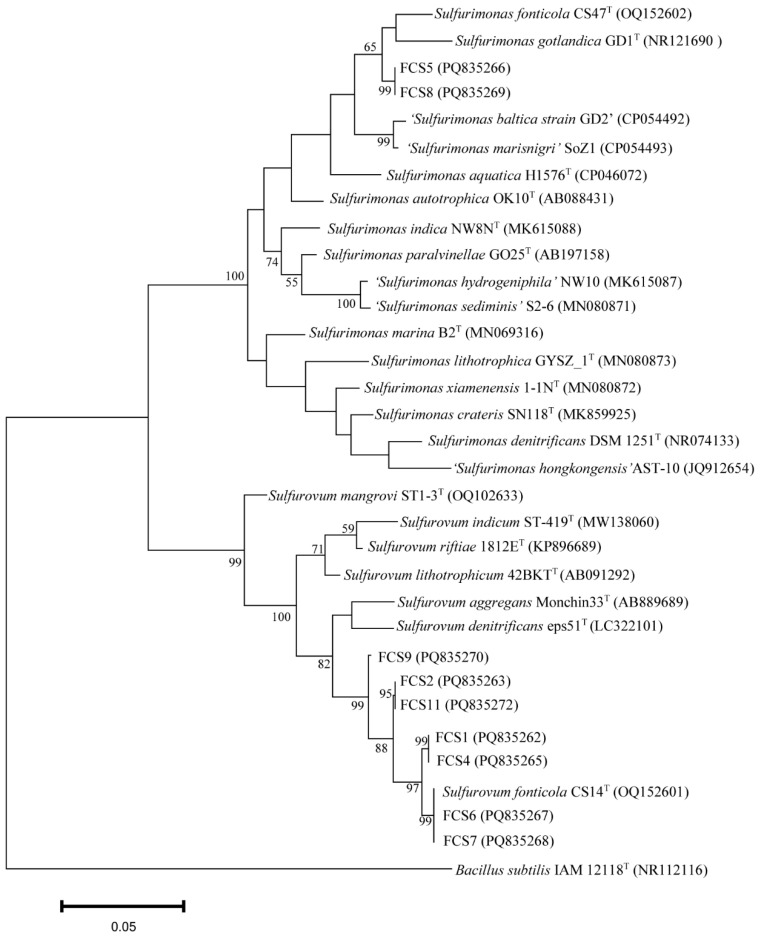
Phylogenetic analysis of strains FCS1, 2, 4, 5, 6, 7, 8, 9, and 11. The maximum likelihood phylogenetic tree based on the 16S rRNA gene sequence (1240bp) shows the positions of the FCS strains and representatives of other related taxa. *Bacillus subtilis* IAM12118^T^ (GenBank accession number NR112116) was used as an outgroup. Scale bar, 0.05 substitution per nucleotide position.

Since, as shown above, *Sulfurovum* and *Sulfurimonas* had the highest diversities in the cold seep, we aimed to isolate these strains from the various samples. To date, *Sulfurovum fonticola* CS14 and *Sulfurimonas fonticola* CS47 are the only strains isolated from cold seep [15]. CS14 was able to grow in the MJH medium (MJ medium with hydrogen) but with a long lag phase (could be more than 17 days) (Figure 2A; Table S3). The presence of sulfur markedly promoted the growth of *Sulfurovum* but had no effect on the growth of *Sulfurimonas* (Figure 2A; Table S3). This optimized medium (i.e., MJH with S^0^) was then used to enrich the Formosa cold seep samples. Eleven enrichments were obtained (Figure 2B). Subsequent 16S rRNA sequence analysis showed that *Sulfurovum* and *Sulfurimonas* were indeed enriched (Figure 2C). In total, nine strains were isolated and named FCS (Formosa Cold Seep) 1, 2, 4, 5, 6, 7, 8, 9, and 11 (Table S4). Based on 16S rDNA and phylogenetic analyses, seven strains were classified under the genus *Sulfurovum* (Figure 3 and Table S4). Strains FCS2 and 11 were 100% identical and most closely related to *S. fonticola* CS14 (98.4% identity). Strain FCS9 was also most closely related to *S. fonticola* CS14 (97.8% identity) (Figure 3 and Table S4). According to the 98.7% identity threshold of species [34], strains FCS2, 9, and 11 were novel species of the genus *Sulfurovum*. Strains FCS6 and FCS7 were 100% identical to *S. fonticola* CS14, and strains FCS1 and FCS4 were most closely related (99.1% identity) to *S. fonticola* CS14, implying that these strains may belong to *S. fonticola* (Figure 3 and Table S4). FCS5 and FCS8 were classified as *Sulfurimonas* and were most closely related (96.7% identity) to *Sulfurimonas fonticola* CS47 (Figure 3 and Table S4). Hence, these two strains are novel species of the genus *Sulfurimonas*.

### 3.3. Characterization of Two Novel Campylobacterota Species

#### 3.3.1. Morphological and Physiological Characterization

**Figure 4:**
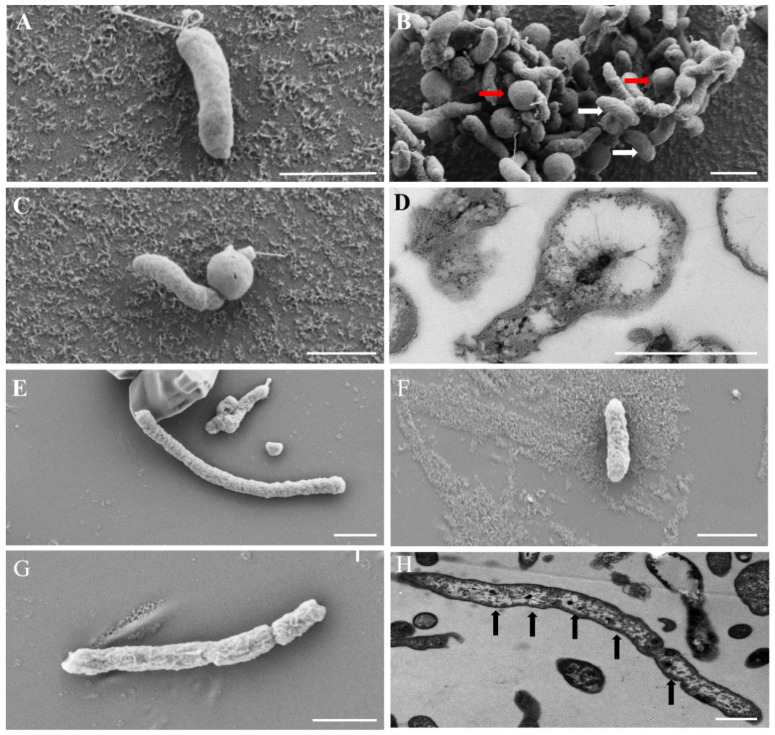
Electron micrographs of the cells of strains FCS5 (**A**–**D**) and FCS9 (**E**–**H**). (**A**–**C**) Strain FCS5 observed with an SEM. Red and white arrows indicate spherical and rod-shaped bacterial cells, respectively. (**D**) The ultrathin section of strain FCS5 observed with a TEM. (**E**–**G**) Strain FCS9 observed with a SEM. (**H**) Strain FCS9 observed with a TEM. Black arrows indicate nucleoids. For all panels, bar, 1 μm.

Strains FCS5 and FCS9, which, as shown above, are potentially novel species of *Sulfurimonas* and *Sulfurovum*, respectively, were selected for further characterization. FCS5 exhibited Gram-negative staining and facultative anaerobic characteristics. It possessed a single polar flagellum and had a width ranging from 0.3 to 0.5 μm and a length of 1.0–2.0 μm. Under SEM, FCS5 cells were mainly rod- or spherical-shaped (Figure 4A,B), but some cells appeared to be rods with a bulging spherical head (Figure 4C). Similar light-bulb structures were detected using TEM examination of the ultrathin sections of FCS5 (Figure 4D). Strain FCS9 was Gram-negative and anaerobic. It measured 0.3–0.5 μm in width and 1.0–2.0 μm in length. Some FCS9 cells surpassed 10 μm in length. No flagellum was observed with FCS9. Under SEM, FCS9 was visualized as filamentous or rod-shaped bacteria (Figure 4E,F), and some bacterial cells were apparently in the process of division (Figure 4G). TEM examination of the ultrathin sections of FCS9 revealed multiple nucleoids in long filamentous bacteria (Figure 4H). A growth study showed that FCS5 could grow within a temperature range of 5–20 °C, a salinity range of 1–3% (*w*/*v*), and a pH range of 5.5–7.5. The optimal growth conditions were 20 °C, 1% NaCl (*w*/*v*), and pH 6.5 (Table 1). These results suggested that strain FCS5 was psychrotolerant. The doubling time of FCS5 was approximately 28 h. When nitrate served as the electron acceptor, FCS5 was able to grow with H_2_, elemental sulfur, and thiosulfate as the electron donors (Table 1). When hydrogen was the electron donor, FCS5 could grow with nitrate or oxygen (not exceeding 5%) as the electron acceptor (Table 1). Heterotrophic growth experiments demonstrated that FCS5 was chemoautotrophic and could not utilize the tested organic compounds (Table 1). For strain FCS9, it grew within a temperature range of 5–25 °C, a salinity range of 2–4% (*w*/*v*), and a pH range of 6.0–8.5. FCS9 exhibited optimal growth under the condition of 15 °C, 3% NaCl (*w*/*v*), and pH 6.5 (Table 1). These results suggested that strain FCS9 was psychrophilic. The doubling time of FCS9 was approximately 84 h. When nitrate was used as the electron acceptor, FCS9 grew chemoautotrophically only with H_2_ as the electron donor and did not grow with sulfide, thiosulfate, sulfite, ditetrasulfate, or S^0^ as the electron donors (Table 1). With hydrogen as the electron donor, FCS9 could grow with nitrate and S^0^ as the electron acceptors but could not grow with thiosulfate or oxygen as the electron acceptor (Table 1). Heterotrophic growth indicated that strain FCS9 did not grow in the tested organic compounds (Table 1), suggesting that it was chemoautotrophic.

**Figure 5:**
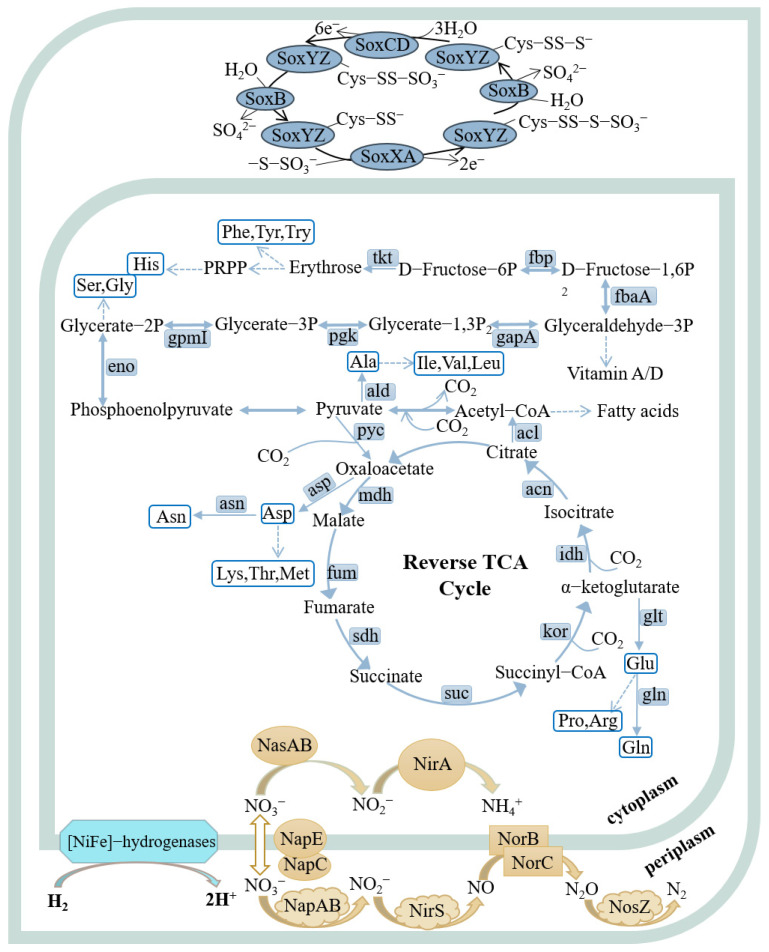
Metabolic pathways of strains FCS5 and FCS9 were reconstructed based on the KEGG database. In the pathway diagrams, metabolic routes are depicted by solid arrows, and dashed lines signify multi-step processes. Strains FCS5 and FCS9 are similar in the examined metabolic pathways, except that FCS5 possesses a complete sulfur-oxidizing system (*soxABXYZ* and *soxCDYZ*), while FCS9 possesses only *soxCDYZ*.

**Figure 6:**
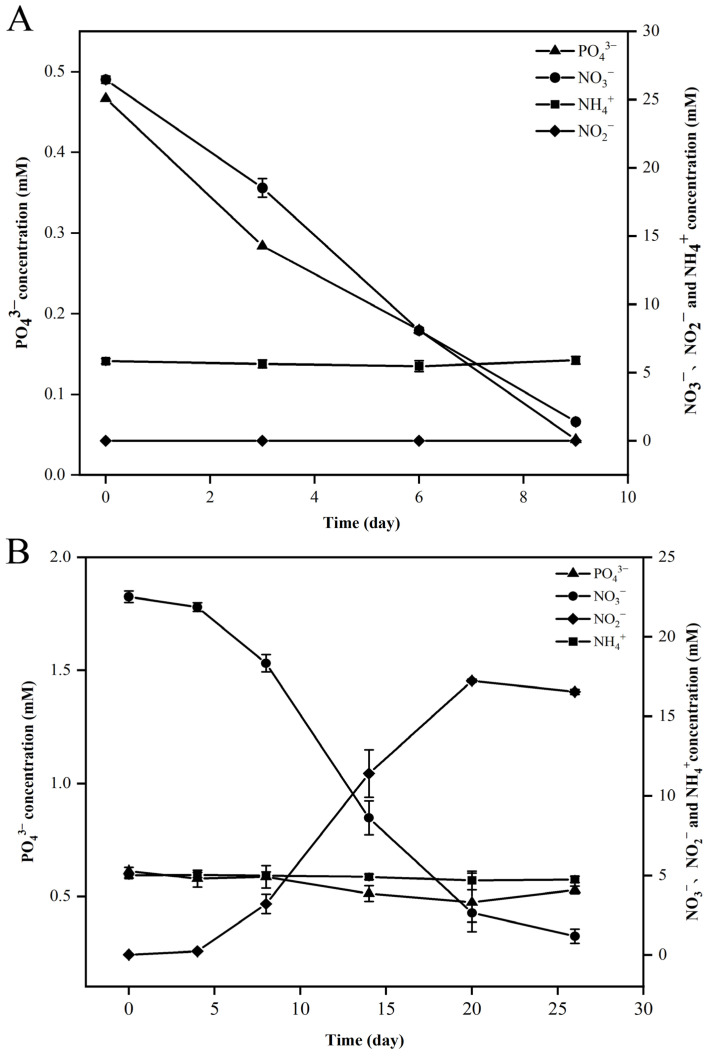
Changes in nitrate (NO_3_^−^), nitrite (NO_2_^−^), ammonia (NH_4_^+^), and phosphate (PO_4_^3−^) during the growth of strains FCS5 (**A**) and FCS9 (**B**). Values are the means of triplicate experiments and shown as means ± SD.

Genomic analysis revealed that strains FCS5 and FCS9 possessed genes associated with hydrogen oxidation, the rTCA (reverse tricarboxylic acid) pathway, and the denitrification pathway (Figure 5, Table S5). Strain FCS5 possessed an intact sulfur-oxidizing system, namely *soxABXYZ* and *soxCDYZ*. In contrast, strain FCS9 harbored a truncated sulfur-oxidizing system consisting solely of *soxCDYZ* (Figure 5, Table S5). Through Raman spectroscopy, consumptions of H_2_ and CO_2_, as well as the production of N_2_, by strains FCS5 and FCS9, were detected, providing direct evidence for the metabolic activities of these strains (Figure S1). To further investigate the growth characteristics of FCS5 and FCS9, the changes in the chemical compositions of their culture medium were examined. The results showed that for both strains, the nitrate content in the medium decreased during bacterial growth (Figure 6A,B). However, while a marked nitrite accumulation was observed with FCS9, no apparent nitrite accumulation was observed with FCS5 (Figure 6A,B), suggesting that FCS5 possessed an efficient denitrification mechanism that completely converted nitrate to other products. In addition to nitrate, phosphate also decreased sharply in the medium of FCS5 (Figure 6A), implying an ability of FCS5 to consume phosphate. Moreover, three type 2 family polyphosphate kinase enzymes and one RNA degradosome polyphosphate kinase enzyme were identified in the genome of strain FCS5, indicating a potential for polyphosphate anabolism and catabolism.

#### 3.3.2. Phylogenomic and Chemotaxonomic Characterization

Genomic examination revealed that strain FCS5 harbored a circular chromosome comprising 2,837,546 base pairs and 2874 predicted genes. Strain FCS9 possessed a circular chromosome of 2,864,372 base pairs with 2892 predicted genes. The G + C contents of the FCS5 and FCS9 genomes were 33.1% and 37.8%, respectively (Table 1). The Average Nucleotide Identity (ANI) values and the digital DNA-DNA Hybridization (DDH) values between strains FCS5 and FCS9 and their closely related members of *Sulfurimonas* and *Sulfurovum*, respectively, are detailed in Table S6. For both strains, the ANI and DDH values are significantly lower than the threshold levels established for prokaryotic species demarcation, i.e., 95–96% for ANI and 70% for DDH [34,35]. Fatty Acid Methyl Ester (FAME) analysis showed that the predominant fatty acids (≥10%) in strain FCS5 encompassed summed feature 3, C_16:0_, and C_14:0_ (Table S7). For strain FCS9, the major fatty acids (≥10%) consisted of summed feature 3, summed feature 8, and C_16:0_ (Table S7), which were similar to that of the reference strains. Collectively, these results indicated that strains FCS5 and FCS9 represented novel species of the genera *Sulfurimonas* and *Sulfurovum*, respectively, and were designated *Sulfurimonas iocasae* sp. nov and *Sulfurovum iocasae* sp. nov, respectively.

### 3.4. The Nutrient Composition of Autotrophic Campylobacterota

To detect their nutrient components, autotrophic Campylobacterota bacteria were subjected to analysis of vitamins, fatty acids, free amino acids, and total proteins. The results showed that strains FCS5, FCS9, CS14, and CS47 did not require vitamins for growth (Table 1). We then examined the vitamins that were produced by FCS5. Fourteen vitamins were identified, of which vitamin A (retinol) had the highest content (21,750.7 ± 2257.7 ng/g) (Table 2). Other identified vitamins included riboflavin, pyridoxine, nicotinamide, thiamine, pantothenic acid, pyridoxal 5′-phosphate, nicotinic acid, folic acid, pyridoxal, cyanocobalamin, biotin, 25-hydroxyvitamin D3 and 25-hydroxyvitamin D2 (Table 2). Since, to our knowledge, vitamins A and D had not been detected previously in Campylobacterota, we explored the molecular basis for the vitamins A/D production observed in our study. Genome search discovered that the 1-deoxy-d-xylulose-5-phosphate (DOXP) pathway was conserved in all examined Campylobacterota members (Table S8). This pathway is responsible for the biosynthesis of isopentenyl diphosphate and dimethylallyl diphosphate, which are precursors of vitamins A and D. Vitamin A commonly functions as the cofactor of rhodopsins in microbes [36]. However, no rhodopsin gene was found in the genome of strain FCS5. To examine the effect of vitamins on global gene expression, the transcriptome profiles of FCS5 grown in the presence (V+) and absence (V−) of vitamins were analyzed. The results showed that the V+ and V− groups were clustered into two distinct clades (Figure S2A). Between the V+ and V− groups, 2520 differentially expressed genes were identified. Specifically, in the V− group, 1255 and 1265 genes were upregulated and downregulated, respectively (Figure S2B). Of the upregulated genes, 17 were involved in thiamine, biotin, pantothenate, and CoA biosynthesis (Table S9), and the genes associated with the synthesis of ribosomes and some amino acids were significantly enriched (Figure S2C). Most of the enzymes in the rTCA pathway were also enriched (Figure S2C). In contrast, in the V+ group, ABC transporters and sugar metabolism genes were significantly enriched (Figure S2D). Since all of the amino acid metabolic pathways are complete or nearly complete in the genome of FCS5 (Figure 5), we measured the amino acid contents in the bacterial cells. Eighteen amino acids were detected, of which glutamate had the highest content (1023.4 ± 29.1 μg/g). Other amino acids with high contents included glycine (889.8 ± 16.1 μg/g) and alanine (856.4 ± 72.8 μg/g) (Table S10). Additionally, we also measured the fatty acids in bacterial cells. Acetic acid was the predominant short-chain fatty acid (486.7 μg/g), palmitoleic acid (6181.8 ± 1142.9 μg/g), palmitic acid (4104.4 ± 971.2 μg/g), and myristic acid (998.8 ± 258.4 μg/g) were the most abundant medium and long-chain fatty acids (Table S10). Protein analysis indicated that the protein content of strains FCS5 and CS14 were 61.4 ± 1.6% and 54.9 ± 3.9%, respectively, which were higher than that of *Methylocystis parvus* [37] but lower than that of *Cupriavidus necator* [38] and *Clostridium autoethanogenum* [39] (Table S11).

## 4. Discussion

In this study, a high-throughput methodology was used to explore the diversity of Campylobacterota in a deep-sea cold seep. Altogether, six families were discovered, all of which fall within the previously documented mesophilic group [3]. Remarkably, no representatives of thermophilic families were uncovered, implying that temperature played an important role in shaping the distribution of Campylobacterota. With respect to diversity, 1091 ASVs were detected, predominately *Sulfurovum* and *Sulfurimonas*. Nearly 100 ASVs, however, defied classification at the genus/family level, signifying the existence of plenty of hitherto uncharacterized novel species in cold-seep Campylobacterota. By means of optimizing the culturing technique, we succeeded in isolating nine strains, including two novel species. These strains were able to grow under low-temperature conditions, and one was psychrophilic. These results indicated that the cold seep harbored a wide and diverse variety of Campylobacterota, with a sizeable fraction being novel psychrophilic/psychrotolerant bacteria. Furthermore, we found that sulfur could act as a stimulant for the growth of Campylobacterota autotrophs, especially *Sulfurovum*. Since elemental sulfur is abundant in the animal communities of the Formosa cold seep and distributed in the same ecological niche as the autotrophic Campylobacterota [28,40], it may enhance the productivity of the autotrophic Campylobacterota. Similar situations might exist in hydrothermal vents, as elemental sulfur is also rich in hydrothermal fluids [41]. Hence, the optimized approach developed in this study may potentially be applied to the isolation of Campylobacterota autotrophs from cold seeps, hydrothermal vents, and other similar habitats containing reduced sulfur. Of the isolated Campylobacterota in our study, both *Sulfurovum* and *Sulfurimonas* exhibited unique morphological features. Strain FCS9 appeared as filamentous structures of varying lengths with multiple nucleoid centers. This may be the result of asymmetric division as that reported for *Helicobacter*, which forms multinucleated cells during division [42]. A previous study showed that in many hydrothermal vent mats, *Sulfurovum* predominantly exists in a long rod-like shape that seemingly gives the bacteria an advantage in the in situ environments [43]. Unlike FCS9, strain FCS5 was morphologically characterized by light-bulb structures, the cause of which remains to be studied.

In hydrothermal vents, chemoautotrophic Campylobacterota shares an intimate relationship with animals by serving as a significant food source for the latter [24]. This is likely also the case in the Formosa cold seep, where Campylobacterota coexist with animals in the same habitats [28]. In the present work, we observed abundant chemoautotrophic Campylobacterota in the setae of *S. crosnieri*, suggesting that these bacteria might be a dietary supplement for the host animal. Microorganisms rich in proteins can be utilized as single-cell proteins, which have been applied to aquaculture feed [44]. In this study, we found that autotrophic Campylobacterota contained a high level of proteins, accounting for 50–60% of the cell dry weight, and a variety of free amino acids, implying an ability of these bacteria to serve as protein providers in the cold seep. Fatty acids are essential for all organisms. We found that autotrophic Campylobacterota were rich in acetic acid, palmitoleic acid, palmitic acid, and myristic acid, the latter three being vital constituents of cell membranes and occurring in marine invertebrates like mussel and shrimp [45,46]. Since these three fatty acids make up more than 10% of the dry weight of the Campylobacterota bacteria, they may supplement the host’s demand for fatty acids. Vitamins are indispensable for all organisms, as they are extensively implicated in diverse biochemical reactions [47,48]. For instance, riboflavin plays a pivotal part in the enzymatic reactions that underpin energy metabolisms [49]. However, animals lack the capacity to synthesize riboflavin and thus must procure it from external sources [50]. Vitamin A is fundamental to the growth, reproduction, and visual function of animals [51]. Similar to the case of riboflavin, animals cannot synthesize vitamin A from scratch and have to depend on the provision of precursor substances like carotene [52]. In this study, we discovered for the first time that autotrophic Campylobacterota could grow without externally sourced vitamins and were competent in synthesizing as many as 14 different vitamins, in particular vitamin A and riboflavin. This result suggests that these bacteria may constitute a significant source of vitamins for deep-sea animals.

Many photosynthetic microorganisms are capable of producing vitamin A, which serves as the chromophore of rhodopsins that participate in light absorption [36]. Although we detected vitamin A in Campylobacterota, no rhodopsin-encoding genes were found in the genome, suggesting that vitamin A functions in cellular processes not requiring rhodopsin. This is reasonable, given that there is no light in the deep sea. Intriguingly, the isoprenoid synthesis pathway, responsible for synthesizing the precursor substances of vitamins A and D, was conserved among autotrophic Campylobacterota, indicating that vitamin A and vitamin D were likely essential for the growth/survival of autotrophic Campylobacterota. Since autotrophic Campylobacterota are frequently distributed at the redox interface of deep-sea environments, such as cold seeps and hydrothermal vents, and some are presumed to live in environments with a high oxygen concentration [53], vitamin A, being an antioxidant [54,55], likely plays an important role in the oxygen toleration ability of these bacteria. In the absence of added vitamins, the bacteria increased the expressions of the pathways of carbon fixation and protein/amino acid/vitamin synthesis. Considering that many intermediates of the pathways serve as substrates for vitamin synthesis, the enhanced expressions of these pathways probably enabled the bacteria to maintain growth by synthesizing the required vitamins.

Some *Sulfurovum* and *Sulfurimonas* are denitrifying bacteria and play an important role in the nitrogen cycle of ecosystems [15,56]. These bacteria are considered to be usable in autotrophic denitrification systems, and indeed, *Sulfurovum* and *Sulfurimonas* have been detected in autotrophic nitrogen removal systems [57,58,59]. In this study, we found that the isolated cold seep *Sulfurovum* (FCS9) and *Sulfurimonas* (FCS5) also exhibited denitrification ability, suggesting an application potential in nitrogen removal. To date, only one strain of Campylobacterota, i.e., *Sulfurimonas gotlandica* GD1^T^, was reported to be able to remove phosphorus [60]. In the present study, we found that strain FCS5 could eliminate not only nitrate but also phosphate. Consistently, the genes involved in polyphosphate anabolism and catabolism were identified in the genome of strain FCS5. These findings indicate a role of autotrophic Campylobacterota in the phosphorus cycle of deep-sea cold seep and that FCS5 may be a good candidate for removing nitrogen and phosphorus in wastewater systems. In addition to wastewater treatment, autotrophic Campylobacterota may also be applied to other situations. Currently, human-induced atmospheric carbon dioxide represents a principal driver of global climate change, which demands the development of carbon-negative manufacturing technologies. Chemoautotrophic microbes have the capacity to convert gaseous one-carbon (C1) waste into renewable chemicals and fuels, thus presenting an attractive option for recycling waste carbon into useful materials [61]. Given that autotrophic Campylobacterota can convert C1 to valuable bioproducts, such as proteins, vitamins, and fatty acids, they potentially hold promise not only as single-cell proteins/vitamins but also for application in carbon dioxide emission reduction.

In conclusion, this study analyzed the diversity and metabolic characteristics of cold seep Campylobacterota. This analysis is limited to just one cold seep and a small number of strains. In the future, more cold seeps and a larger number of strains may be explored.

Description of *Sulfurimonas iocasae* sp. nov (i.o.ca’sae. N.L. fem. gen. n. iocasae name arbitrarily formed from IOCAS, the acronym for Institute of Oceanology, Chinese Academy of Sciences, where the study on strain FCS5^T^ was carried out).

Cells are Gram-negative, facultative anaerobic, 1.0–2.0 μm long and 0.3 to 0.5 μm wide, motile by a polar flagellum. Grows occur at 5–20 °C (optimum 20 °C), pH 5.5–7.5 (optimum 6.5), and 1–3% (wt/vol) NaCl (optimum 1% [wt/vol]). Obligate chemolithoautotrophic growth occurs with hydrogen, elemental sulfur, or thiosulfate as the electron donors and nitrate or oxygen (not exceeding 5%) as the electron acceptor. Organic substrates are not utilized as carbon sources. The genomic DNA G + C content is 33.1 mol%.

The type strain, FCS5^T^ (=MCCC 1K09359^T^ = KCTC 25901^T^), was isolated from the deep-sea cold seep in the SCS.

Description of *Sulfurovum iocasae* sp. nov (i.o.ca’sae. N.L. fem. gen. n. iocasae name arbitrarily formed from IOCAS, the acronym for Institute of Oceanology, Chinese Academy of Sciences, where the study on strain FCS9^T^ was carried out).

Cells are Gram-negative, non-motile, and anaerobic, 0.3–0.5 μm wide and 1.0–2.0 μm long. Growth occurs at 5–25 °C (optimum 15 °C), pH 6.0–8.5 (optimum 6.5), and 2–4% (wt/vol) NaCl (optimum 3% [wt/vol]). Obligate chemolithoautotrophic growth occurs with hydrogen as the sole electron donor and nitrate or elemental sulfur as the electron acceptor. Organic substrates are not utilized as carbon sources. The genomic DNA G + C content is 37.8 mol%.

The type strain, FCS9^T^ (=CGMCC 1.18167^T^), was isolated from the deep-sea cold seep in the SCS.

## Figures and Tables

**Table 1:** Comparison of the characteristics of strains FCS5 and FCS9 and the related species. 1, FCS5; 2, *Sulfurimonas fonticola* CS47^T^ [15]; 3, FCS9; 4, *Sulfurovum fonticola* CS14^T^ [15]. *, Data from this study; ND, Not determined; +, Positive; −, Negative.

Characteristics	1 *	2	3 *	4
Shape	Rod to slightly curved	Rod to slightly curved	Rod or filament	Ellipsoid or rod
Temperature range (optimal) (°C)	5–20(20)	0–37(25–28)	5–25(15)	5–20(10)
pH range(optimal)	5.5–7.5(6.5)	6.5–7.5(6.5)	6.0–7.5(6.5)	5.5–8.5(6.5)
NaCl range (optimal) (%)	1–3(1)	1–6(2–3)	2.0–4.0(3.0)	2.0–4.0(3.0)
Electron donors	H_2_, S^0^, S_2_O_3_^2−^	H_2_, S^0^ *	H_2_	H_2_ *
Heterotrophic growth	–	– *	–	– *
Organic electron donors	–	–*	–	– *
Electron acceptors	NO_3_^−^,O_2_	NO_3_^−^, O_2_*	NO_3_^−^, S^0^	NO_3_^−^ *
Vitamin dependent	–	– *	–	– *
G + C content (%)	33.1	32.1	37.8	37.6

**Table 2:** Vitamins detected in strain FCS5. DCW, dry cell weight.

Vitamin	ng/g DCW	Vitamin	ng/g DCW
Water-Soluble		Fat-Soluble	
Riboflavin	3546.1 ± 2004.1	Retinol	21,750.7 ± 2257.7
Pyridoxine	2275.7 ± 1301.2	25-Hydroxyvitamin D3	117.4 ± 19.4
Nicotinamide	1708.2 ± 232.7	25-Hydroxyvitamin D2	22.3 ± 2.3
Thiamine	601.8 ± 214.1		
Pantothenic acid	217.6 ± 20.8		
Pyridoxal 5′-phosphate	73.2 ± 37.0		
Nicotinic acid	55.6 ± 36.8		
Folic acid	44.7 ± 12.7		
Pyridoxal	37.6 ± 24.7		
Cyanocobalamin	5.7 ± 0.5		
Biotin	1.7 ± 1.7		

## Data Availability

The amplicon sequencing data were deposited in the Short Reads Archive (National Center for Biotechnology Information) under the BioProject number PRJNA1206233. The complete genome sequences of strains FCS5 and FCS9 were deposited in GenBank under the BioProject numbers PRJNA1206339 (accession number CP180409) and PRJNA1206344 (accession number CP180410), respectively. The transcriptomic sequences were deposited in the Short Reads Archive under the BioProject number PRJNA1206546.

## Supplementary Materials

The following supporting information can be downloaded at: https://www.mdpi.com/article/10.3390/microorganisms13051028/s1, Table S1: Summary of the sequencing information of the samples used in this study; Table S2: Distribution of Campylobacterota at the ASV level and OTU level (97%); Table S3: Optimization of the medium with different electron acceptor(s). +, grow; −, not grow; Table S4: Strains isolated from the cold seep in this study; Table S5: Carbon and energy metabolism of strains FCS5 and FCS9 based on genome analysis. +, positive; −, negative; Table S6: The ANI and DDH values of strains FCS5 and FCS9 with other members of *Sulfurimonas* and *Sulfurovum*; Table S7: Cellular fatty acid compositions of FCS5 and FCS9 and other members of *Sulfurovum* and *Sulfurimonas*. 1, FCS5; 2, *Sulfurimonas fonticola* CS47^T^; 3, FCS9; 4, *Sulfurovum fonticola* CS14^T^; Fatty acids that represent < 0.5% in all columns are omitted. Fatty acids that represent > 5.0% are in bold. −, Not detected; Table S8: Distribution of the 1-deoxy-d-xylulose-5-phosphate (DOXP) pathway in autotrophic Campylobacterota.1, FCS5; 2, FCS9; 3, *Sulfurimonas fonticola* CS47; 4, *Sulfurovum fonticola* CS14; 5, *Sulfurovum denitrificans* eps51^T^; 6, *Sulfurovum lithotrophicum* 42BKT^T^; 7, *Sulfurimonas hydrogeniphila* NW10^T^; 8, *Sulfurimonas gotlandica* GD1^T^; 9, *Nautilia profundicola* AmH^T^; 10, *Nitratifractor salsuginis* E9I37-1^T^; 11, *Hydrogenimonas leucolamina* SS33^T^; 12, *Desulfurella amilsii* TR1^T^; Table S9: The upregulated genes associated with vitamin biosynthesis in the V− group; Table S10: Amino acids and fatty acids detected in strain FCS5 with metabolomics. DCW, dry cell weight; Table S11: Protein content in FCS5, CS14, and other chemoautotrophic strains; Figure S1: Raman spectra of CO_2_, N_2_, and H_2_ detected during the growth of strains FCS5 and FCS9. The peak areas are indicated in brackets. The numerical values represent the relative contents, and the decrease/increase in the numerical values indicates that the substance is being consumed/produced; Figure S2: Transcriptome analysis of strain FCS5 cultured in the presence (V+) and absence (V−) of vitamins. (A) Clustering analysis of three V− samples and three V+ samples. (B) Volcano plot analysis of differential gene expression between V− and V+ groups. (C,D) KEGG enrichment analysis of the upregulated genes in the V− (C) and V+ (D) groups.
