# Supplementary material for: Deep-Sea Cold Seep Campylobacterota: Diversity, Growth, Metabolic Characteristics, and Nutrient Production
Source: Microorganisms. 2025 Apr 29;13(5):1028. doi: 10.3390/microorganisms13051028 (PMC12113678; doi:10.3390/microorganisms13051028)

Supplemental data - methods

Transcriptome analysis

Strain FCS5 was cultured in MJH medium supplemented with or without vitamins to the late logarithmic late stage. Total RNA was extracted from the cells with a RNA extract kit (Tiangen, Beijing, China) and used for mRNA purification. First strand cDNA was synthesized using random hexamer primers and M - MuLV reverse transcriptase, followed by RNase H treatment to degrade the RNA. The first strand cDNA was then used for synthesizing the second strand cDNA. The remaining overhangs were converted into blunt ends via exonuclease/polymerase activities. After adenylation of the 3' ends of the DNA fragment, adaptors with a hairpin loop structure were ligated to the DNA. Then USER Enzyme was used to degrade the second strand cDNA. To select cDNA fragments with a length of 370 - 420 bp, the library fragments were purified with the AMPure XP system (Beckman Coulter, Beverly, USA). Subsequently, PCR was performed with Phusion High - Fidelity DNA polymerase, universal PCR primers, and Index (X) primers. Finally, the PCR product was purified (AMPure XP system), and the library quality was assessed on the Agilent Bioanalyzer 2100 system. The clustering of the index - coded samples was performed on a cBot Cluster Generation System using the TruSeq PE Cluster Kit v3 - cBot - HS (Illumina, San Diego, CA, USA) according to the manufacturer's instructions. After cluster generation, the library preparations were sequenced on an Illumina Novaseq platform, and 150 bp paired - end reads were generated. The raw data in fastq format were first processed through perl scripts. In this step, clean data were obtained by removing reads containing adapters, N base, and low-quality reads from the raw data. The Q20, Q30, and GC contents of the clean data were calculated. HTSeq v0.6.1 was used to count the reads numbers mapped to each gene. The FPKM (expected number of fragments per kilobase of transcript sequence per million base pairs sequenced) of each gene was calculated based on the length of the gene and the reads count mapped to this gene. Differential expression analysis was performed using the DESeq R package (1.18.0). The resulting P - values were adjusted using the Benjamini and Hochberg's approach for controlling the false discovery rate. Gene Ontology (GO) enrichment analysis of differentially expressed genes was implemented with the GOseq R package, in which gene length bias was corrected. GO terms with corrected P - value less than 0.05 were considered significantly enriched by differential expressed genes. The KOBAS software was used to test the

statistical enrichment of pathways.

Metabolomic analysis of short-chain fatty acids

Strain FCS5 was cultured in MJH medium at the optimum temperature and harvested at the late logarithmic stage. The cells were washed two times with ultrapure water and dried. The sample preparation for metabolomic analysis was as follows: (1) 1 ml NaOH solution (5 mmol/L) was added into 50 mg sample that had been precooled at 4 °C; (2) the mixture was homogenized for 3 minutes (with the sample tray pre-cooled at -20 °C) and then ultrasonically extracted for 7 minutes in an ice bath; (3) the mixture was centrifuged at 12,000 rpm for 10 minutes at 4 °C, and 500 μl supernatant was transferred into an injection vial, followed by adding 300 μl pure water. Sample derivatization was then performed as follows: (1) 500 μl propanol/pyridine (3:2, v/v) and 100 μl propyl chloroformate were added into the injection vial, followed by vortexing for 10 seconds and then sonication for 1 minute; (2) 300 μl n-hexane was added into the injection vial and vortexed at 2000 rpm for 60 seconds; (3) after centrifugation at 12,000 rpm for 5 minutes at 4 °C, 250 μl of the n-hexane layer was transferred into a new injection vial; (4) 200 μl n-hexane was continuously added into the original injection vial, followed by vortexing at 2000 rpm for 60 seconds; (5) after centrifugation at 12,000 rpm for 5 minutes at 4 °C, 200 μl of the n-hexane layer was transferred into the new injection vial; (6) 10 mg anhydrous sodium sulfate was added into 450 μl n-hexane layer, followed by vortexing or 10 seconds, and then the sample was analyzed using a GC-MS system. Standards derivatization was conducted as follows: (1) 300 μl of mixed standards solution and 500 μl NaOH solution (5mM) were added into an injection vial; (2) 500 μl propanol/pyridine (3:2, v/v) and 100 μl propyl chloroformate were added into the injection vial, followed by vortexing for 10 seconds and then sonication for 1 minute; (3) 300 μl n-hexane was added into the injection vial (vial A), followed by vortexing at 2000 rpm for 60 seconds; (4) after centrifugation at 12,000 rpm for 5 minutes at 4 °C, 250 μl of the n-hexane layer was transferred into a new injection vial (vial B); (5) 200 μl n-hexane was continuously added into the injection vial A, followed by vortexing at 2000 rpm for 60 seconds; (6) after centrifugation at 12,000 rpm for 5 minutes at 4 °C, 200 μl of the n-hexane layer was transferred into the injection vial B; (7) 10 mg of anhydrous sodium sulfate was added into the 450 μl n-hexane layer of vial B , followed by vortexing for 10 seconds, and then the sample was analyzed using a gas chromatography-mass spectrometry (GC-MS) system. A 5977B

GC/MSD (with CTC Automatic sampler) was used. The chromatographic conditions were as follows: an HP-5MS capillary column (30 m × 0.25 mm × 0.25 μm, Agilent J&W Scientific, Folsom, CA, USA); high-purity helium (with a purity not less than 99.999%) as the carrier gas; a flow velocity of 1.0 mL/minute; and an injection port temperature of 260 °C. One microliter sample was injected in split mode with a split ratio of 10:1 and a solvent delay of 2.2 minutes. The temperature programming was as follows: the column oven temperature was initial set at 50 °C and maintained for 5.2 minutes, then increased to 70 °C at a rate of 10 °C/minute and maintained for 1.3 minutes, then increased to 85 °C at a rate of 3 °C/minute and maintained for 1 minute, then increased to 110 °C at a rate of 5 °C/minute and maintained for 1 minute, and finally increased to 290 °C at a rate of 30 °C/minute and maintained for 9 minutes. The mass spectrometry conditions were as follows: an electron bombardment ion source, with an ion source temperature of 230 °C, a quadrupole temperature of 150 °C, and an electron energy of 70 eV. The scanning mode was full scan mode, and the quality scan range was set at 30 to 600 m/z.

Metabolomic analysis of medium and long chain fatty acids

The samples were resuspended in glass centrifuge tubes with 1 mL of chloroform/methanol (at a volume ratio of 1:2) and lysed through sonication. The supernatant was collected and mixed with 2 mL of H_2SO_4/methanol solution (with a volume ratio of 1:99) at 80 °C for the purpose of methyl esterification. The resulting methyl esters were extracted using 1 mL of hexane. Immediately prior to analysis, 25 μL of nonadecanoic acid methyl ester was added as an internal standard into each 500 μL of the extracted sample or the mixed standard solution. Gas chromatography-mass spectrometry (GC-MS) was carried out by employing an Agilent 7890 gas chromatograph coupled with an Agilent 5975C mass spectrometer. The chromatography was performed using a high-resolution gas chromatography capillary column (with a length of 30 m, an inner diameter of 0.25 mm, and coated with 0.25 μm dimethyl polysiloxane, provided by Agilent Technologies). The temperature programming was as follows: an initial hold at 50 °C for 3 minutes, followed by a gradual increase to 220 °C at a rate of 10 °C per minute, and finally a hold at 220 °C for 5 minutes. Helium was utilized as the carrier gas at a flow rate of 1.0 mL/min. The injection volume was set at 1 μL, with a split injection ratio of 1:10. This was done to assess the stability and reproducibility of the detection system. The mass spectrometry conditions were as follows: the injection port, ion

source, and transfer line temperatures were set to 280 °C, 230 °C, and 250 °C respectively. The electron bombardment was the electron impact ionization source with an ionizing energy of 70 eV, operating in the selected ion monitoring scanning mode. The mass data were analyzed using the MSD ChemStation software (from Agilent Technologies) to extract the chromatographic peak area and retention time. Finally, a standard curve was generated, and the individual fatty acid concentration of each sample was calculated. The complete platform and the fatty acid methyl ester mixed standard solutions were furnished by Shanghai Applied Protein Technology Company, Ltd.

Metabolomic analysis of vitamins

The samples were thawed in an ice bath and then added to a 96-well plate after mixing with stock standards. To each sample, 100 mL of cold methanol was added, and the mixture was vigorously vortexed for 5 minutes. The plate was then centrifuged at 4000g for 30 minutes, and 30 mL of the supernatant was transferred to a new 96-well plate. Then 20 mL of freshly prepared 3-nitrophenylhydrazine and 1-ethyl-3-(3-dimethyl aminopropyl) carbodiimide was added to each well. The plate was sealed, and the derivatization process was carried out at 30 °C for 60 minutes. After derivatization, 80 mL of cold 50% methanol was added to dilute the sample. The plate was then stored at -20 °C for 20 minutes and centrifuged at 4000g at 4 °C for 30 minutes. From each well, 120 mL of the supernatant was transferred to a new 96-well plate. Finally, the plate was sealed for LC-MS analysis. A Shimadzu ultra-performance liquid chromatography coupled to QTrap 6500+ (AB Sciex corporation, California, USA) was used to quantify the targeted metabolites. A BEH C18 column (with dimensions of 2.1 × 100 mm and a particle size of 1.7 mm) was utilized, and the following gradient was applied: from 0 to 1 minute, 5% B; from 1 to 4 minutes, 5 - 25% B; from 4 to 8 minutes, 25 - 50% B; from 8 to 10 minutes, 50 - 100% B; from 10 to 12 minutes, 100% B; from 12 to 12.1 minutes, 100 - 5% B; from 12.1 to 15 minutes, 5% B. Mobile phase A was 0.1% formic acid in water, and B was acetonitrile. The flow rate was set at 0.35 mL/min. The column temperature was maintained at 40 °C, and the injection volume was 3 mL. The peak area integration and quantification of the targeted metabolites were performed using the SCIEX OS software 1.0 (AB Sciex Corp.).

Metabolomic analysis of free amino acids

To extract metabolites from the sample, 400 μl of a cold methanol and acetonitrile (1:1, v:v) extraction solvent was added to eliminate the protein and extract the metabolites. Then, the mixture was thoroughly vortexed. For absolute quantification of the metabolites, stock solutions of stable-isotope internal standards were simultaneously added to the extraction solvent. The mixture was then transferred into a new centrifuge tube and centrifuged at 13,800g for 20 minutes at 4 °C to collect the supernatant. The supernatant was dried in a vacuum centrifuge. For LC-MS analysis, the sample was redissolved in 100 μl acetonitrile:water (1:1, v:v) solvent and centrifuged at 13,800g at 4 °C for 20 minutes. Subsequently, the supernatant was injected. The analysis was carried out using an UHPLC (1290 Infinity LC, Agilent Technologies) coupled to a QTRAP MS (AB 6500+, AB Sciex). The metabolites were separated using hydrophilic interaction chromatography (Waters UPLC BEH Amide column, with dimensions of 2.1 × 100 mm and a particle size of 1.7 μm) and C18 columns (Waters UPLC BEH C18x2.1 × 100 mm, 1.7 μm). The metabolites were quantified using 6500+ QTRAP (AB Sciex) in MRM mode and in positive and negative switch mode.

Supplemental data - Tables

Table S1. Summary of the sequencing information of the samples used in this study.

Sample	Total Tags	Shannon	Simpson	Goods_coverage (%)	ASVs
SS1	62976	7.09	0.93	100	2455
SS2	55140	8.44	0.98	100	2388
SS3	72481	7.48	0.96	100	2069
SS4	59757	8.91	0.98	100	2626
SS5	66182	8.44	0.98	100	2085
SS6	59203	9.32	0.99	100	2587
SS7	56517	9.47	0.99	100	2607
SS8	78714	5.42	0.88	100	1166
SS9	67955	7.88	0.97	100	2181
SS10	72469	8.60	0.98	100	2036
SS11	65537	8.40	0.98	100	2044
SS12	76873	7.61	0.96	100	1821
FS1	47370	5.40	0.93	100	271
FS2	62768	6.23	0.96	100	361
FS3	60822	5.91	0.94	100	291
FS4	49603	4.64	0.85	100	249
AS1	54861	4.83	0.90	100	152
AS2	58249	4.53	0.90	100	131
AS3	58888	4.72	0.91	100	162
AS4	52729	5.31	0.95	100	174

Table S2. Distribution of Campylobacterota at the ASV level and OTU level (97%).

Order	Family	Genus	ASV	OTU
Campylobacterales	Sulfurovaceae	*Sulfurovum*	793	140
		unidentified	5	3
	Sulfurospirillaceae	*Sulfurospirillum*	2	2
	Sulfurimonadaceae	*Sulfurimonas*	154	35
		unidentified	2	1
	Helicobacteraceae	*Helicobacter*	8	3
		unidentified	6	1
	Campylobacteraceae	*Campylobacter*	2	2
	Arcobacteraceae	*Arcobacter*	34	6
		unidentified	1	2
	Unidentified		84	30

Table S3. Optimization of the medium with different electron acceptor(s). +, grow; -, not grow.

Strain	Medium	Dilution	2d	4d	6d	9d	13d	15d	17d	21d
CS14	MJH	10^{-1}	-	-	-	-	-	-	-	+
		10^{-2}	-	-	-	-	-	-	-	-
		10^{-3}	-	-	-	-	-	-	-	-
	MJH with S^0	10^{-1}	-	+	+	+	+	+	+	+
		10^{-2}	-	-	+	+	+	+	+	+
		10^{-3}	-	-	-	+	+	+	+	+
	MJH with $Na_2S_2O_3$	10^{-1}	-	-	-	+	+	+	+	+
		10^{-2}	-	-	-	-	-	-	-	-
		10^{-3}	-	-	-	-	-	-	-	-
	MJH with S^0 and $Na_2S_2O_3$	10^{-1}	-	+	+	+	+	+	+	+
		10^{-2}	-	-	+	+	+	+	+	+
		10^{-3}	-	-	-	+	+	+	+	+
CS47	MJH	10^{-1}	+	+	+	+	+	+	+	+
		10^{-2}	-	+	+	+	+	+	+	+
		10^{-3}	-	+	+	+	+	+	+	+
	MJH with S^0	10^{-1}	+	+	+	+	+	+	+	+
		10^{-2}	-	+	+	+	+	+	+	+
		10^{-3}	-	+	+	+	+	+	+	+
	MJH with $Na_2S_2O_3$	10^{-1}	+	+	+	+	+	+	+	+
		10^{-2}	-	+	+	+	+	+	+	+
		10^{-3}	-	+	+	+	+	+	+	+
	MJH with S^0 and $Na_2S_2O_3$	10^{-1}	+	+	+	+	+	+	+	+
		10^{-2}	-	+	+	+	+	+	+	+
		10^{-3}	-	+	+	+	+	+	+	+

Table S4. Strains isolated from the cold seep in this study.

Strain	Source	The most closely related type strain	Identity
FCS1	Sediment	*Sulfurovum fonticola* CS14^{T}	99.1%
FCS2	*Alvinocaris* shrimp gills	*Sulfurovum fonticola* CS14^{T}	98.5%
FCS4	Sediment	*Sulfurovum fonticola* CS14^{T}	99.5%
FCS5	*Shinkaia crosnieri* setae	*Sulfurimonas fonticola* CS47^{T}	96.7%
FCS6	Sediment	*Sulfurovum fonticola* CS14^{T}	100%
FCS7	Sediment	*Sulfurovum fonticola* CS14^{T}	100%
FCS8	Sediment	*Sulfurimonas fonticola* CS47^{T}	96.7%
FCS9	Sediment	*Sulfurovum fonticola* CS14^{T}	97.8%
FCS11	*Shinkaia crosnieri* setae	*Sulfurovum fonticola* CS14^{T}	98.4%

Table S5. Carbon and energy metabolism of strains FCS5 and FCS9 based on genome analysis. +, positive; –, negative.

	FCS5	FCS9
**Sulfur oxidation**		
Sox	SoxCDYZ，SoxABXYZ;	SoxCDYZ
Sulfide:quinone oxidoreductas	+	+
**Hydrogen oxidation**		
[NiFe]-Hydrogenases	+	+
**Nitrogen metabolism**		
Dissmillatory nitrate reduction	–	–
Assimillatory nitrate reduction	+	+
Denitrification	+	+
Nitrogen fixation	+	–
**Carbon fixation** (rTCA)		
ATP citrate lyase	+	+
Oxoglutarate:ferredoxin oxidoreductase (Oor)	+	+
Pyruvate:ferredoxin oxidoreductase	+	+

Table S6. The ANI and DDH values of strains FCS5 and FCS9 with other members of *Sulfurimonas* and *Sulfurovum*.

Strain	FCS5 (ANI/DDH)
Sulfurimonas gotlandica GD1^{T}	83.21%/26.60%
Sulfurimonas fonticola CS47	81.63/25.30%
Sulfurimonas hongkongensis AST-10^{T}	76.94%/20.80%
Candidatus Sulfurimonas marisnigri SoZ1	76.24%/21.20%
Candidatus Sulfurimonas baltica GD2	75.65%/21.10%
Sulfurimonas xiamenensis 1-1N^{T}	74.46%/20.00%
Sulfurimonas aquatica H1576^{T}	73.85%/20.10%
Sulfurimonas denitrificans DSM 1251^{T}	73.82%/19.80%
Sulfurimonas lithotrophica GYSZ_1^{T}	73.46%/19.50%
Sulfurimonas crateris SN118^{T}	72.86%/18.40%
Sulfurimonas autotrophica DSM 16294^{T}	72.80%/ 19.00%
Sulfurimonas indica NW8N^{T}	72.31%/19.20%
Sulfurimonas marina B2^{T}	72.11%/19.40%
Sulfurimonas sediminis S2-6^{T}	72.06%/20.00%
Sulfurimonas hydrogeniphila NW10^{T}	71.85%/20.10%
Sulfurimonas paralvinellae GO25^{T}	71.77%/19.20%
Strain	FCS9 (ANI/DDH)
Sulfurovum fonticola CS14^{T}	90.70%/42.40 %
Sulfurovum xiamenensis XTW-4	76.19%/20.70%
Sulfurovum denitrificans eps51T	74.98%/20.80%
Sulfurovum lithotrophicum ATCC BAA-797	73.31%/19.50%
Sulfurovum riftiae 1812E	72.93%/19.40%
Sulfurovum indicum ST-419	72.78%/19.50%
Sulfurovum zhangzhouensis zt1-1	72.56%/19.40%
Sulfurovum mangrove ST1-3	72.40%/20.70%

Table S7. Cellular fatty acid compositions of FCS5 and FCS9 and other members of *Sulfurovum* and *Sulfurimonas*. 1, FCS5; 2, *Sulfurimonas fonticola* CS47^T; 3, FCS9; 4, *Sulfurovum fonticola* CS14^T; Fatty acids that represent < 0.5% in all columns are omitted. Fatty acids that represent > 5.0% are in bold. –, Not detected.

Fatty acid	1	2	3	4
**Saturated**				
$C_{12:0}$	0.25	0.09	**3.56**	**3.16**
$C_{14:0}$	**11.22**	**7.19**	**7.94**	**8.24**
$C_{16:0}$	**15.46**	**22.91**	**11.10**	**9.64**
**Unsaturated**				
$C_{16:1}$ ω5c	–	–	1.12	1.63
**Summed features:†**				
2(12:0 aldehyde)	**5.09**	**6.61**	**5.73**	**5.56**
3($C_{16:1}$ ω7c/ ω6c)	**55.64**	**49.40**	**51.95**	**52.61**
5($C_{18:0}$ ante/ $C_{18:2}$ ω6,9c)	–	–	0.96	0.97
8($C_{18:1}$ ω7c/ ω6c)	**9.29**	**9.35**	**17.64**	**17.98**

Table S8. Distribution of the 1-deoxy-d-xylulose-5-phosphate (DOXP) pathway in autotrophic Campylobacterota.1, FCS5; 2, FCS9; 3, *Sulfurimonas fonticola* CS47; 4, *Sulfurovum fonticola* CS14; 5, *Sulfurovum denitrificans* eps51^{T}; 6, *Sulfurovum lithotrophicum* 42BKT^{T}; 7, *Sulfurimonas hydrogeniphila* NW10^{T}; 8, *Sulfurimonas gotlandica* GD1^{T}; 9, *Nautilia profundicola* AmH^{T} ; 10, *Nitratifractor salsuginis* E9I37-1^{T} ; 11, *Hydrogenimonas leucolamina* SS33^{T}; 12, *Desulfurella amilsii* TR1^{T}.

DOXP pathway	1	2	3	4	5	6	7	8	9	10	11	12
1-Deoxy-D-xylulose-5-phosphate synthase	+	+	+	+	+	+	+	+	+	+	+	+
1-Deoxy-D-xylulose-5-phosphate reductoisomerase	+	+	+	+	+	+	+	+	+	+	+	+
2-C-methyl-D-erythritol 4-phosphate cytidylyltransferase	+	+	+	+	+	+	+	+	+	+	+	+
4-Diphosphocytidyl-2-C-methyl-D-erythritol kinase	+	+	+	+	+	+	+	+	+	+	+	+
(E)-4-hydroxy-3-methylbut-2-enyl-diphosphate synthase	+	+	+	+	+	+	+	+	+	+	+	+
2-C-methyl-D-erythritol 2,4-cyclodiphosphate synthase	+	+	+	+	+	+	+	+	+	+	+	+
4-Hydroxy-3-methylbut-2-en-1-yl diphosphate reductase	+	+	+	+	+	+	+	+	+	+	+	+

Table S9. The upregulated genes associated with vitamin biosynthesis in the V− group.

No.	Gene name	log_2Fold change	p-Value	Padj
	**Thiamine metabolism**			
1	Phosphomethylpyrimidine synthase	2.57	7.23609E-46	3.5785E-45
2	Cysteine desulfurase	4.07	3.52245E-60	2.9199E-59
3	Thiazole synthase	1.16	2.09999E-13	3.7758E-13
4	Thiamine-phosphate pyrophosphorylase	1.92	3.69149E-30	1.1148E-29
5	Thiamine-monophosphate kinase	1.39	1.83112E-10	3.0031E-10
	**Pantothenate and CoA biosynthesis**			
6	Acetolactate synthase	3.45	5.39744E-78	1.048E-76
7	Ketol-acid reductoisomerase	3.06	8.46188E-49	4.5843E-48
8	Dihydroxy-acid dehydratase	2.77	3.36941E-65	3.5706E-64
9	3-Methyl-2-oxobutanoate hydroxymethyltransferase	1.30	2.96884E-10	4.8525E-10
10	Branched-chain amino acid aminotransferase	3.09	2.73034E-65	2.9147E-64
11	Dephospho-CoA kinase	2.51	6.9539E-48	3.6644E-47
12	Pantetheine-phosphate adenylyltransferase	2.30	1.25295E-42	5.6024E-42
13	Aspartate 1-decarboxylase	2.95	1.10878E-49	6.1568E-49
	**Biotin metabolism**			
14	3-Oxoacyl-[acyl-carrier-protein] synthase II	2.55	7.3014E-40	2.9501E-39
15	3-Oxoacyl-[acyl-carrier protein] reductase	1.24	2.8769E-15	5.5338E-15
16	3-Hydroxyacyl-[acyl-carrier-protein] dehydratase	2.64	2.05709E-45	1.0104E-44
17	Enoyl-[acyl-carrier protein] reductase I	1.37	3.99936E-20	8.8864E-20

Table S10. Amino acids and fatty acids detected in strain FCS5 with metabolomics. DCW, dry cell weight.

Metabolite	Weight (μg/g DCW)
**Amino acid**	
Glutamate	1023.4±29.1
Glycine	889.8±16.1
Alanine	856.4±72.8
Tyrosine	280.2±106.0
Lysine	229.2±36.3
Glutamine	163±22.5
Valine	68.3±1.5
Histidine	51.5±5.3
Arginine	44.6±3.1
Leucine	31.9±1.5
Phenylalanine	30.0±0.7
Methionine	26.5±2.5
Isoleucine	20.7±1.2
Serine	20.2±2.0
Tryptophan	11.5±1.0
Threonine	10.3±0.6
Proline	9.1±1.0
Asparagine	8.6±5.9
**Short-chain fatty acid**	
Acetic acid	486.7±49.8
Propionic acid	2.8±0.4
Hexanoic acid	1.1±0.3
Isobutyric acid	0.7±0.1
Butyric acid	0.6±0.0
Valeric acid	0.6±0.1
Isovaleric acid	0.2±0.0
**Medium and long- chain fatty acid**	
Palmitoleic acid	6181.8±1142.9
Palmitic acid	4104.4±971.2
Myristic acid	998.8±258.4
Pentadecanoic acid	58.5±29.6
Stearic acid	55.0±4.3
10Z-Heptadecenoic acid	28.1±28.4
Myristoleic acid	27.1±8.3
Heptadecanoic acid	19.6±9.3
Lauric acid	12.9±4.1
Oleic acid	4.9±3.4
10Z-Pentadecenoic acid	4.7±2.2
8Z,11Z,14Z-Eicosatrienoic acid	3.8±0.3
Tridecanoic acid	2.7±1.2

Linolelaidic acid	1.8±0.5
Linoleic acid	1.6±0.5
11Z-Eicosenoic acid	1.3±0.0
Decanoic acid	1.0±0.4
Erucic acid	1.0±0.6
11Z,14Z,17Z-Eicosatrienoic acid	0.9±0.1
Heneicosanoic acid	0.6±0.2
Adrenic acid	0.5±0.1
Octanoic acid	0.5±0.2
Arachidic acid	0.5±0.1
Elaidic acid	0.5±0.2
13Z,16Z-Docosadienoic acid	0.3±0.3
Nervonic acid	0.3±0.2
α-Linolenic acid	0.3±0.1
Lignoceric acid	0.2±0.0
Behenic acid	0.1±0.1
11Z,14Z-Eicosadienoic acid	0.1±0.1
γ-Linoleic acid	0.1±0.0
4Z,7Z,10Z,13Z,16Z-Docosapentaenoic acid	0.1±0.0
4Z,7Z,10Z,13Z,16Z,19Z-Docosahexaenoic acid	0.1±0.0

Table S11. Protein content in FCS5, CS14, and other chemoautotrophic strains.

Strain	Protein content	Reference
FCS5	61.4±1.6%	This study
CS14	54.9±3.9%	This study
Methylocystis parvus OBBP	37.1 %	[35]
Cupriavidus necator	75.6%	[36]
Clostridium autoethanogenum	80%	[37]

Supplemental data - Figures

Figure S1. Raman spectra of CO_2, N_2, and H_2 detected during the growth of strains FCS5 and FCS9. The peak areas are indicated in brackets. The numerical values represent the relative contents, and the decrease/increase in the numerical values indicates that the substance is being consumed/produced.

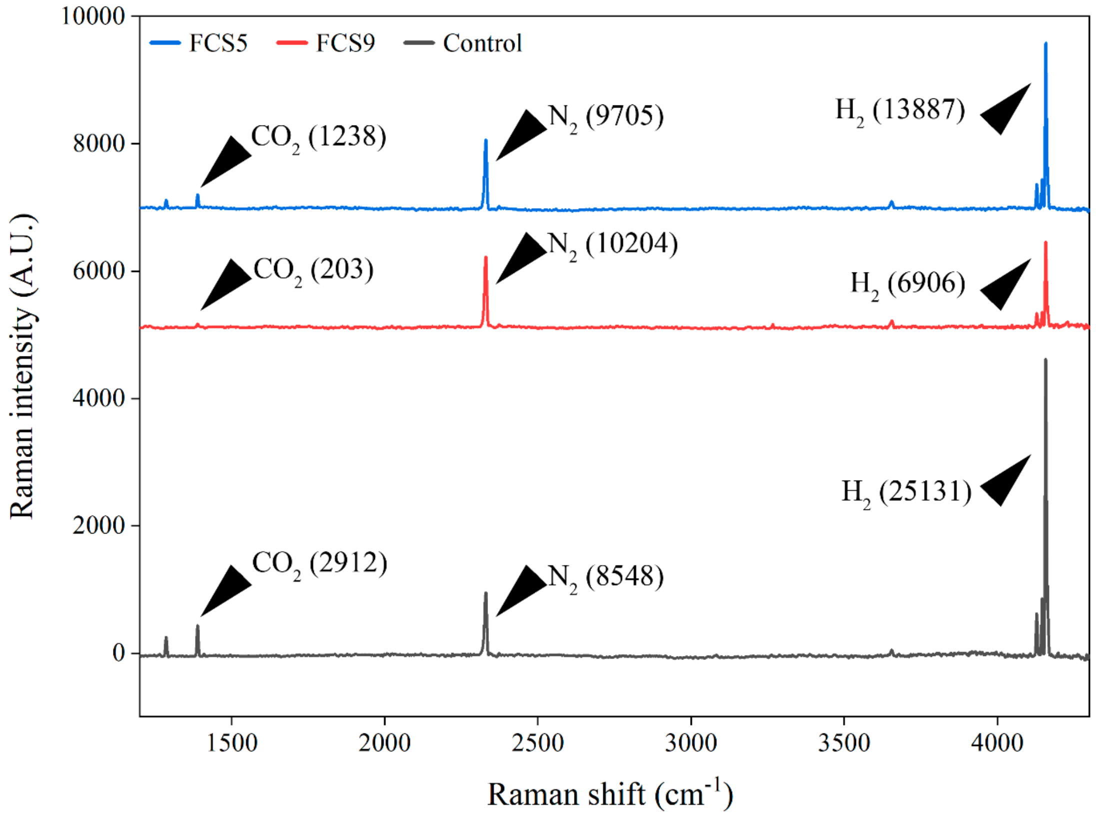

Figure S2. Transcriptome analysis of strain FCS5 cultured in the presence (V+) and absence (V−) of vitamins. (**A**) Clustering analysis of three V− samples and three V+ samples. (**B**) Volcano plot analysis of differential gene expression between V− and V+ groups. (**C,D**) KEGG enrichment analysis of the upregulated genes in the V− (C) and V+ (D) groups.

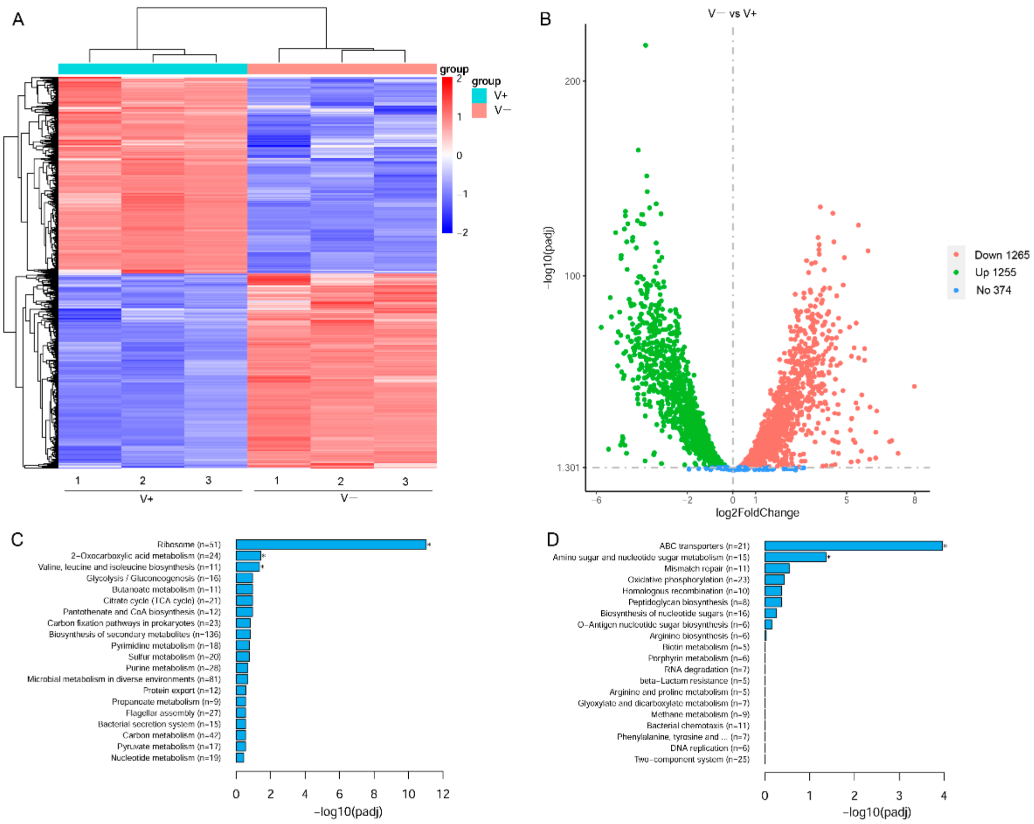

Supplement: Supplementary file 1 [file microorganisms-13-01028-s001.zip › microorganisms-3563659-supplementary.pdf]
